# Retrospective Analysis of Coronavirus SARS-CoV-2 Antibody Levels in COVID-19 Convalescent Plasma From Blood Donors

**DOI:** 10.1155/cjid/4366502

**Published:** 2024-12-05

**Authors:** Ji He, Yanjun Zhang, Jie Dong, Wei Zhang, Faming Zhu

**Affiliations:** ^1^Institute of Transfusion Medicine, Blood Center of Zhejiang Province, Jianye Road 789, Hangzhou, Zhejiang 310052, China; ^2^Zhejiang Provincial Centers for Disease Control and Prevention, Bingsheng Road 3399, Hangzhou, Zhejiang 310052, China

**Keywords:** antibody, COVID-19, SARS-CoV-2

## Abstract

**Objective:** To detect and analyze coronavirus SARS-CoV-2 antibody levels in convalescent plasma from donors who have recovered from COVID-19.

**Methods:** Plasma samples from 88 donors aged 20–54 years who were diagnosed with COVID-19 and who were eligible to donate from Zhejiang Province, China, were collected as the experimental group, and 56 samples from healthy blood donors were used as controls. Anti-SARS-CoV-2 antibodies, including Ab and IgM, were detected via chemiluminescent immunoassay, and neutralizing antibodies were measured via the microneutralization method.

**Results:** The positive rates of total and IgM antibodies against SARS-CoV-2 were 97.7% (86/88) and 52.3% (46/88), respectively, in the plasma samples of 88 patients who recovered from COVID-19. After 160 and 320 dilutions of the total antibody-positive samples, the positive rates were 61.6% (53/86) and 39.5% (34/86), respectively. The titer of neutralizing antibodies was 16–256 in 53 SARS-CoV-2-positive samples after 160-fold dilution of total antibodies. The titer of neutralizing antibody was 48–256 in 34 samples that were still positive after 320-fold dilution of total antibody. Among the 88 samples, 86 had titers > 4, and 10 had high titers > 80. In 2 patients with neutralizing antibody titers < 4, SARS-CoV-2 total antibody and IgM antibodies were negative. The correlation coefficient between total antibody strength and neutralizing antibody titer was 0.5198 (high correlation). The total antibody and IgM antibodies of 56 healthy blood donors were negative.

**Conclusions:** There are individual differences in plasma antibody titers among convalescent patients. Antibody detection is helpful for screening out plasma with high antibody titers for the treatment of patients with severe disease.

## 1. Introduction

Severe acute respiratory syndrome coronavirus 2 (SARS-CoV-2) emerged in late 2019 in China and was declared a pandemic by the World Health Organization (WHO) in March 2020. As of September 2021, over 200 million individuals have been infected with COVID-19, which has had an immense impact on the health care system worldwide [1]. The COVID-19 pandemic has affected every aspect of life and is still ongoing three years after it began. The world was caught off guard by the COVID-19 pandemic [2]. Currently, some protocols have been explored for the treatment of this disease. One investigational treatment explored for COVID-19 is the use of convalescent plasma (CP) collected from individuals who have recovered from COVID-19 [3–5].

Passive immunization via CP seems to be an attractive treatment option. The plasma collected from recovered individuals contains specific polyclonal antibodies, which can help neutralize the pathogen and increase the recipient's immune response [6]. CP has been studied in outbreaks of other respiratory infections, such as pandemic influenza, SARS and Middle East respiratory syndrome (MERS) [7–10]. Because of the comparable virological and clinical characteristics among SARS, MERS, and COVID-19 patients [11], CP may be effective for the treatment of COVID-19. Some reports have shown that plasma therapy can effectively reduce the symptoms and mortality of patients with SARS-CoV-2 infection when there is no specific treatment for COVID-19 [3, 12, 13], and CP transfusion has shown good safety results in hospitalized patients with COVID-19 [14]; however, the mechanism of action of CP is not fully understood, as factors such as antibody functional activity [15, 16], antibody specificity, affinity, and host immune features, including endogenous antibody levels, may influence its effectiveness.

SARS-CoV-2 antibody levels are highly correlated with neutralizing titers [17, 18]. Neutralizing antibodies are antibodies that target the surface antigens of a virus and can bind to free viruses in the body, preventing the virus from being absorbed and invading cells. IgM, IgG, and IgA antibodies have neutralizing activity, while IgG antibodies, especially spike (S) protein receptor binding domain (RBD)-specific IgG, exist for a long time after recovery because of their high content and specificity in body fluids. A study of plasma therapy in 5 patients with severe COVID-19 suggested that plasma donors were selected to meet the following conditions: serum SARS-CoV-2-specific IgG antibody titer greater than 1:1000 (ELISA) and neutralizing antibody titers greater than 40 [3]. The patient treated by CP with high titers of IgG did not require mechanical ventilation 11 days after plasma transfusion and was then transferred to a general ward [19]. Therefore, the CP antibody titer is the key factor in determining whether it can be used for treatment. In the process of plasma treatment, dynamic screening of specific antibodies is needed for both donors and patients. The National Health Commission of China recommends collecting plasma within 2 weeks after the recovery period [20–22]. Here, plasma from COVID-19 patients who had been discharged for two weeks was collected from our blood center, after which total antibodies, IgM and neutralizing antibodies against SARS-CoV-2 were detected. Our serological findings in CP from recovered patients may facilitate the understanding of antibodies and establish a CP donor screening protocol for the COVID-19 outbreak.

## 2. Materials and Methods

### 2.1. Donors

The plasma samples of 88 patients who had recovered from COVID-19 two weeks previously were obtained and mobilized from the Zhejiang Blood Center to collect CP, all of which met the conditions of the “Clinical Treatment Plan for CP of Patients Recovered from COVID-19 (Trial Version 2)” issued by the National Health Commission of China. The inclusion criteria for CP donors were as follows: (1) Convalescent patients who were diagnosed with COVID-19 by nucleic acid tests and met the discharge and release isolation criteria of the “Diagnosis and Treatment Plan for COVID-19 (Trial Version 6)” [23] issued by the National Health Commission of the People's Republic of China; (2) patients who were discharged from the hospital and isolated for more than 2 weeks; (3) patients aged 18–55 years; (4) patients whose body weight was ≥ 50 kg for males or ≥ 45 kg for females; (5) patients with no blood-transmitted diseases; and (6) patients whose plasma could be donated after evaluation by clinicians.

The control samples were from 56 healthy unpaid blood donors from Zhejiang Blood Center. All the samples were retained with informed consent. This study was approved by the Ethical Committee of Zhejiang Blood Center. Written informed consent was obtained from the participants.

The collected blood samples were centrifuged at 1500 to 2000x g for 10** **min at 4°C, and the serum was stored in 2 mL frozen tubes at −20°C.

### 2.2. SARS-CoV-2 RNA Detection

SARS-CoV-2 RNA was detected via real-time fluorescence polymerase chain reaction (PCR) according to the manufacturer's instructions (BioGerm, Shanghai, China). In brief, total RNA was isolated from nasopharyngeal swabs, after which one-step reverse transcription PCR (including the ORF1ab and N genes) was performed for SARS-CoV-2 RNA detection. The result is assigned according to the Ct value and PCR amplification curve.

### 2.3. SARS-CoV-2 Total Antibody and IgM Antibody Detection

The total antibody and IgM antibody against SARS-CoV-2 in the plasma samples were tested via chemiluminescence microparticle immunoassay (CMIA) kits and a Caris 2001 chemiluminescence immunoassay analyzer (Xiamen Wantai Kerry Biotechnology Co., Ltd., Xiamen, China) according to the manufacturer's instructions. In brief, the sample and reference control were mixed with SARS-CoV-2 recombinant antigen-coated magnetic particles for a certain reaction time, and acridine ester labeled with the SARS-CoV-2 recombinant antigen was then added after washing. If the SARS-CoV-2 antibody is present, it will form a SARS-CoV-2 recombinant antigen-coated magnetic particle/SARS-CoV-2 antibody/SARS-CoV-2 antigen-labeled acridine ester complex. After washing again, pre-excitation and excitation solutions were added, and the generated chemiluminescence reaction signal was measured. The amount of SARS-CoV-2 antibodies present in a sample is positively correlated with the relative light unit (RLU) detected via the Caris system, and the S/CO value of the SARS-CoV-2 IgM or IgG antibody can be automatically calculated on the basis of the RLU and the built-in calibration curve. When the sample S/CO ratio was < 1.0, the SARS-CoV-2 antibody was regarded as nonreactive. When the sample S/CO ratio was ≥ 1.0, the SARS-CoV-2 antibody was considered reactive. In accordance with the CP infusion therapeutic guidelines approved by the National Health Commission of the People's Republic of China, the total antibody-reactive samples were diluted 1:160 and 1:320 in saline and tested via the same method.

### 2.4. Neutralizing Antibody Testing

The neutralizing antibodies were detected via the microneutralization method in the BSL-3 level laboratory at the Zhejiang Provincial Center for Disease Control and Prevention, China. The SARS-CoV-2 virus was isolated from a patient, and Vero cells were cultured according to previous methods [24]. First, the serum was inactivated at 56°C for 30 min and diluted 1:4 to 1:1024 (4-fold serial dilution). The diluted serum was subsequently transferred to a 96-well plate, and the virus was added to each well and mixed. A mixture of 50 *μ*L per well was added to a 96-well plate with a monolayer of Vero cells for 2 h. Moreover, a serum control group, a viral control group and a normal cell control group were established. The cells were cultured in a 5% CO_2_ incubator at 35°C. The virus was propagated in Vero cells and cultured under standard conditions. Cytopathic effects were observed every 24 h, and the distributions of different titer values were recorded. The 50% serum neutralization end point is the highest serum dilution that can protect 50% of cells from lesions, which is the neutralization end point.

### 2.5. Statistical Analysis

The antibody-positive rate was calculated directly. Correlation analysis was performed via GraphPad Prism 5, and *p* < 0.05 was considered statistically significant.

## 3. Results

### 3.1. Characteristics of the Convalescent Donors

The convalescent COVID-19 donors included 40 males and 48 females, with an age range of 20–54 years. All of them were negative for HBsAg, anti-HCV, anti-HIV1/2, and *Treponema pallidum* antibodies, the nucleic acid amplification technology (NAT) for HBV DNA, HCV RNA, and HIV RNA. The plasma samples were negative for SARS-CoV-2 RNA via real-time fluorescence PCR.

### 3.2. Total and IgM Antibodies

A total of 88 COVID-19 convalescent donors and 56 healthy blood donors were tested. The positive rates of total antibody and IgM antibodies in the convalescent donors were 97.7% (86/88) and 52.3% (46/88), respectively. The antibodies of all healthy blood donors were negative. Eighty-six total antibody-reactive samples were diluted 1:160 and 1:320, and the positive rates were 61.6% (53/86) and 39.5% (34/86), respectively (Tables 1 and 2).

### 3.3. Neutralizing Antibodies

**Figure 1:**
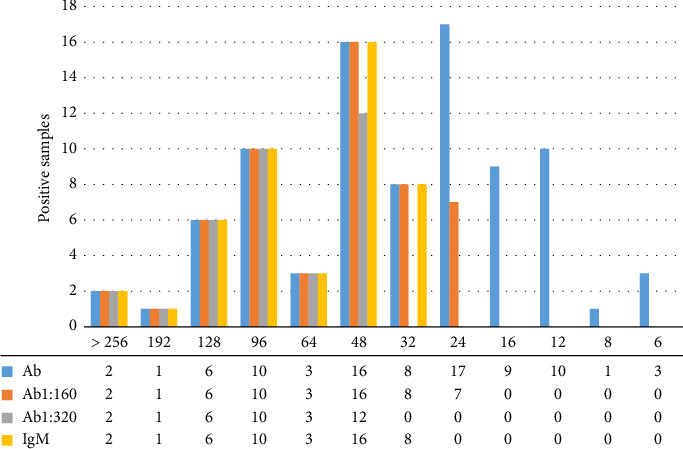
Antibody-positive samples with different neutralizing antibody titers.

Among the 88 patients who recovered from COVID-19, 86 samples had a titer of SARS-CoV-2 neutralizing antibodies > 4, and 10 samples had a titer of neutralizing antibodies > 80, accounting for 11.4% of the total samples. The distributions of total antibody, IgM antibody and total antibody-positive samples diluted 160 and 320 times with different titers of neutralizing antibody are shown in Table 3 and Figure 1. In 2 patients with neutralizing antibody titers < 4, SARS-CoV-2 total antibody and IgM antibodies were negative. The titer of neutralizing antibodies was 16–256 in 53 SARS-CoV-2-positive samples after 160-fold dilution of total antibodies. The titer of neutralizing antibody was 48–256 in 34 samples that were still positive after 320-fold dilution of total antibody. Forty-six SARS-CoV-2 IgM antibody-positive samples had neutralizing antibody titers of 32–256. The neutralizing antibody titers were less than 4 in 56 healthy blood donors.

### 3.4. Correlation Analysis of Total and Neutralizing Antibodies Against SARS-CoV-2

**Figure 2:**
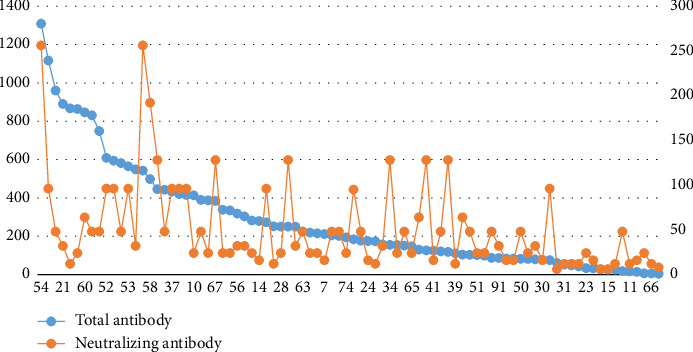
Total antibody COI was correlated with neutralizing antibody titer. The horizontal coordinate is the sample number, the left vertical coordinate is the antibody COI value, and the right vertical coordinate is the neutralizing antibody titer.

The correlation coefficient (*r* = 0.5198; *p* < 0.0001) between the COI values of 86 SARS-CoV-2 total antibody-positive samples and neutralizing antibody titers was highly linear, as shown in Figure 2 (sorted by the AbCOI value).

## 4. Discussion

Previous reports on other viral infections have suggested that CP with higher antibody levels may have a greater effect on the virus load [25–27]. The use of CP therapy involves the use of passive immunotherapy through the high titer of antibodies in the plasma and virus binding to achieve destruction and elimination of the virus. In view of the above experience in the treatment of acute viral infectious diseases with CP and the results of previous studies, it is suggested that CP is also feasible for the treatment of COVID-19. At present, supportive and symptomatic therapy is used for the clinical treatment of COVID-19. In the absence of specific drugs and effective clinical vaccines, CP therapy is currently a better specific clinical treatment [19, 28]. CP therapy has been explored for the treatment of patients with severe COVID-19 in China [29–31]. Plasma should be collected from only selected recovered individuals who have been diagnosed with COVID-19 for at least 3 weeks and who have been isolated for at least 14 days after discharge to minimize the possible risk of SARS-CoV-2 in their blood [32].

According to the laboratory testing provisions of CP in the Clinical Treatment Plan for CP of Patients with COVID-19 (Trial Version 2), in addition to the relevant requirements of the Blood Bank Technical Operating Procedures (2019 version), CP is tested for numerous indicators. In addition to serological and nucleic acid tests for markers of HIV, hepatitis B virus and hepatitis C virus, serological index tests for syphilis antibodies, alanine aminotransferase tests, COVID-19 nucleic acid blood tests and COVID-19 serum/plasma antibody tests are also needed.

When SARS-CoV-2 enters the body, the body has an immune defense effect and produces the specific antibodies IgM, IgG and IgA. The diagnostic index of acute infection is the IgM antibody level, the diagnostic indices of middle and late infection and previous infection are the IgG antibody level, and the total antibody level includes all virus-specific antibodies. Therefore, total antibody detection is highly valuable for both asymptomatic infection and clinical diagnosis [33–35]. Virus-specific antibody detection is an important means for the laboratory diagnosis of viral diseases, and antibody detection is highly important for virus diagnosis and infection time, assessment of the virus infection stage and prediction of disease outcome [36, 37].

The detection of SARS-CoV-2-specific antibodies serves as an indicator of the body's humoral immune response, which is crucial for elucidating therapeutic efficacy and assessing disease outcomes. However, the attenuation and duration of these antibodies remain largely uncharacterized, particularly during the recovery phase. As the virus is completely eradicated, antibody levels significantly decrease. In this study, 86 positive samples were diluted 160 and 320 times, among which the positive rates of total antibody and IgM were 61.6% (53/86) and 39.5% (34/86), respectively. The titers of all the neutralizing antibodies were greater than 4. The other two patients were negative for total antibodies and IgM, with neutralizing antibody titers less than 4. There were only 2 cases with neutralizing antibody titers of 1:266 and 9 cases with antibody titers of 1:288–1:266, accounting for 10.2% of the total samples. The correlation coefficient between the COI values of 86 SARS-CoV-2 total antibody-positive samples and neutralizing antibody titers was highly linear. It is suggested that chemiluminescence can be used to detect total antibody replacement when neutralization detection is not possible. In this study, two cases of total SARS-CoV-2 antibodies in the CP samples of 88 patients with COVID-19 were negative, which may be related to the low content of detection reagents or antibodies in the samples, and further follow-up analysis is needed. According to the “Clinical Treatment Plan for CP of Patients with COVID-19,” the plasma diluted 320 times for the treatment of COVID-19 is still positive, while the results of this experiment revealed that only 39.5% of the collected plasma met this requirement. The COVID-19 Recovery Plasma Industry Guidance issued by the Center for Biologics Evaluation and Research of the U.S. Food and Drug Administration stipulates that the neutralizing antibody titer of recovery plasma should be at least 1:80, and this study showed that only 11.4% of collected plasma met this standard. Due to a lack of clinical data, no further curative effect analysis was performed.

## 5. Conclusions

In conclusion, quantitative antibody detection for convalescent patients and detection of the levels of SARS-CoV-2 antibodies and neutralizing antibodies in CP can be used to screen out volunteers with high titers of antibodies for the preparation of therapeutic CP to ensure the efficacy of CP for patients with severe disease.

## Figures and Tables

**Table 1:** Antibodies from COVID-19 adult donors and healthy blood donors.

Antibody	COVID-19 donors		Healthy blood donors	
	Total antibody	IgM antibody	Total antibody	IgM antibody
Positive	86	46	0	0
Negative	2	42	56	56
Total	88	88	56	56

**Table 2:** Antibodies of diluted reactive samples.

Total antibody dilution	COVID-19 patients		
	Positive	Negative	Total
1:160	53	33	86
1:320	34	52	86

**Table 3:** The neutralizing antibody titers of the total antibody, IgM antibody-positive samples, and diluted samples.

Neutralizing antibody titer	Number	Total antibody		Total antibody 160 dilution		Total antibody 320 dilution		IgM	
		Positive	Negative	Positive	Negative	Positive	Negative	Positive	Negative
> 256	2	2	0	2	0	2	0	2	0
192	1	1	0	1	0	1	0	1	0
128	6	6	0	6	0	6	0	6	0
96	10	10	0	10	0	10	0	10	0
64	3	3	0	3	0	3	0	3	0
48	16	16	0	16	0	12	4	16	0
32	8	8	0	8	0	0	8	8	1
24	17	17	0	7	10	0	17	0	17
16	9	9	0	0	9	0	9	0	9
12	10	10	0	0	10	0	10	0	10
8	1	1	0	0	1	0	1	0	1
6	3	3	0	0	3	0	3	0	3
< 4	2	0	2	—	—	—	—	0	2
Total	88	86	2	53	33	34	52	46	42

## Data Availability

The data used in this study are available from the corresponding author upon reasonable request.
